# Prehospital mSOFA Score for Quick Prediction of Life-Saving Interventions and Mortality in Trauma Patients: A Prospective, Multicenter, Ambulance-based, Cohort Study

**DOI:** 10.5811/westjem.59048

**Published:** 2023-08-08

**Authors:** Francisco Martín-Rodríguez, Ancor Sanz-García, Ana Benito Justel, Almudena Morales Sánchez, Cristina Mazas Perez Oleaga, Irene Delgado Noya, Irene Sánchez Soberón, Carlos del Pozo Vegas, Juan F. Delgado Benito, Raúl López-Izquierdo

**Affiliations:** * Universidad de Valladolid, Faculty of Medicine, Valladolid, Spain; † Emergency Medical Services (SACYL), Advanced Life Support, Valladolid, Spain; ‡ Prehospital Early Warning Scoring-System Investigation Group, Valladolid, Spain; § Universidad de Castilla la Mancha, Faculty of Health Sciences, Talavera de la Reina, Spain; ∥ Hospital Clínico Universitario, Department of Emergency Medicine, Valladolid, Spain; ¶ Hospital Universitario Rio Hortega, Department of Emergency Medicine, Valladolid, Spain; # Universidad Europea del Atlántico, Department of Emergency Medicine, Santader, Spain; ** Universidad Internacional Iberoamericana, Department of Emergency Medicine, Campeche, México; †† Universidade Internacional do Cuanza, Department of Emergency Medicine, Cuito, Bié, Angola

## Abstract

**Background:**

Prehospital emergency medical services (EMS) are the main gateway for trauma patients. Recent advances in point-of-care testing and the development of early warning scores have allowed EMS to improve patient classification. We aimed to identify patients presenting with major trauma involving life-saving interventions (LSI) using the modified Sequential Organ Failure Assessment (mSOFA) score in the prehospital scenario, and to compare these results with those of other trauma scores.

**Methods:**

This was a prospective, ambulance-based, multicenter, training-validation study in trauma patients who were treated in a prehospital setting and subsequently transported to a hospital. The study involved six Advanced Life Support units, 38 Basic Life Support units, and four hospitals. The primary outcome was LSI performed at the scene or en route and intensive care unit (ICU) admission and all-cause two-day in-hospital mortality. We collected epidemiological variables, creatinine, lactate, base excess, international normalized ratio, and vital signs. Discriminative power (area under the receiver operating characteristic curve [AUC]), calibration (observed vs predicted outcome agreement), and decision-curve analysis (DCA, clinical utility) were used to assess the reliability of the mSOFA in comparison to other scores.

**Results:**

Between January 1, 2020–April 30, 2022, a total of 763 patients were selected. The mSOFA score’s AUC was 0.927 (95% confidence interval [CI] 0.898–0.957) for LSI, 0.845 (95% CI 0.808–0.882) for ICU admission, and 0.979 (95% CI 0.966–0.991) for two-day mortality.

**Conclusion:**

The mSOFA score outperformed the other scores, allowing a quick identification of high-risk patients. The routine implementation in EMS of mSOFA could provide critical support in the decision-making process in time-dependent trauma injuries.

Population Health Research Capsule What do we already know about this issue?

*Prehospital identification of major trauma patients requiring life-saving interventions has not yet been elucidated.*

What was the research question?

*Does mSOFA (an early warning score) allow quick identification of high-risk trauma patients?*

What was the major finding of the study? Major comparison with p-value and confidence interval

*The mSOFA score presents an area under the receiver operating characteristic curve of 0.927 (95% CI 0.898–0.957) for life-saving interventions.*

How does this improve population health?

*Using mSOFA to quickly identify high-risk trauma patients could improve treatment and management of these rapidly evolving and complex cases.*

## BACKGROUND

Several complex consequences follow a severe trauma (eg, hypoperfusion, coagulopathy, hypothermia, acidosis, and tissue inflammation). These responses increase morbidity and mortality in trauma patients.^1^ Discriminating major trauma that require life-saving interventions (LSI) vs trauma without systemic repercussions is a particular challenge for emergency medical services (EMS), particularly in prehospital care.^2^^,^^3^ The identification of patients requiring LSI on the scene is critical in deciding whether to transport to the emergency department (ED) of a hospital equipped to manage complex trauma cases (trauma center). Conversely, rapid identification of patients who are not at high risk allows EMS personnel to transport those patients to hospitals with fewer resources and/or clinicians with less training in treating complex trauma. The ability to quickly differentiate between high-risk trauma patients and those not at immediate risk improves the transfer rate of high-complexity cases to trauma centers, thereby optimizing the use of resources. The use of trauma scores to determine patient risk in non-prehospital scenarios has been successfully adopted in clinical practice.^4^

In prehospital care, the usefulness of an early warning score (EWS) has been broadly demonstrated and has become standard practice across numerous EMS agencies.^5^^,^^6^ However, few specific scores are available for EMS application to trauma patients (eg, the revised trauma score [RTS];^7^ new trauma score [NTS];^8^ the combination of mechanism, Glasgow Coma Scale, age, and arterial pressure score [MGAP];^9^ or the Vittel criteria).^10^ However, prehospital point-of-care testing (POCT) has been widely used.^11^ Measurements such as hemoglobin, base excess, pH, lactate, creatinine, or the international normalized ratio (INR) provide relevant feedback, guiding the resuscitation of polytrauma patients from the first moments of EMS arrival on the scene.^12^^,^^13^

The major advantage of using an EWS or POCT, either alone or jointly, is the potential standardization of patient assessment, enabling EMS personnel to recognize high-risk patients without obvious clinical symptoms. On-scene use of diagnostic and/or prognostics tools streamlines the decision-making process. Specifically, these tools provide EMS personnel detailed short-term evolution data that enables them to decide whether to transport the patient to a higher resource facility. Applying EWS in trauma patients also makes it possible to standardize a trauma patient’s management throughout the entire healthcare system. This uniform terminology simplifies patient transfer between clinicians, ultimately lowering the risk of adverse events.^14^^,^^15^

The combined application of physiological parameters and analytical measures has significantly improved the prognostic performance of both EWS and POCT in helping to quickly and decisively identify high-risk patients. A particularly striking example is the Sequential Organ Failure Assessment (SOFA) score, which can discriminate multiorgan damage and is routinely used in intensive care units (ICU).^16^ More recently, the Traumasis-SOFA scoring system is used specifically for patients who have been in a traffic collision.^17^ Despite technological advances, there is no portable POCT capable of measuring bilirubin or platelets in the prehospital setting; thus, prospective estimation of the SOFA score is not currently available at the scene or while en route to the ED. The SOFA score, which combines physiological measurements (Glasgow Coma Scale [GCS], mean arterial pressure [MAP], and pulse oximetry saturation/fraction of inspired oxygen ratio [SaFi]) with analytical determinations (creatinine and lactate), was developed to be used at the scene or en route.^18^ In their 2010 study, Grissom et al proposed the use of a modified SOFA (mSOFA) score,^19^ which was specifically designed and validated for prehospital care, including physiological and analytical parameters available bedside.

In this investigation, our primary aim was to evaluate the effectiveness of prehospital mSOFA scores for predicting the need for LSI (invasive mechanical ventilation, and/or administration of tranexamic acid and/or noradrenaline) in trauma patients. Secondary aims were to explore the performance of mSOFA in predicting ICU admissions and two-day in-hospital mortality, and to compare the mSOFA with four trauma scores (RTS, NTS, MGAP, and the BIG [base deficit, INR, and GCS] score).

## METHODS

### Study Design and Settings

This was a prospective, ambulance-based, multicenter, training-validation study of trauma patients who were treated in the prehospital setting and subsequently transported to an ED. The study involved six Advanced Life Support (ALS) units, 38 Basic Life Support (BLS) units, and four EDs. All the facilities—distributed over three provinces (covering inner city areas, suburbs, and rural areas)—are part of the Public Health System of Castilla y León (Spain), with a reference population of 995,137 inhabitants. The BLS units are staffed by two emergency medical technicians (EMT), and the ALS units are composed of an emergency registered nurse (ERN), a physician, and two EMTs.

Between January 1, 2020–April 30, 2022, patient data was collected from two back-to-back prospective studies conducted under an identical operative guideline. The institutional review board of the Public Health Service reviewed and approved the investigation. The study was registered in the WHO International Clinical Trials Registry Platform (ISRCTN48326533 and ISRCTN49321933); we followed the Transparent Reporting of a Multivariable Prediction Model for Individual Prognosis or Diagnosis (TRIPOD)^20^ guidelines (Supplementary data p3).

### Participants

We included in this study adult trauma patients (>18 years) who were screened by the ALS physician and evacuated by ALS or BLS units to the ED. Only cases with venous line and subsequent blood analysis performed at the scene or en route were included in the follow-up cohort.

Exclusion criteria included the following: cardiorespiratory arrest not recovered at the scene; pregnant women; potential danger to staff; discharged in situ (after evaluation by the ALS physician); or inability to obtain informed consent at the site, en route, or at the ED.

### Score Selection

The scores compared with the mSOFA fulfilled the following conditions: they have been validated; they are available in the prehospital scope of care; and they are based on the determination of standard vital signs or clinical observations and/or a basic blood test. The exclusion criteria included the following: scores with an already known modest predictive capacity (eg, shock index^21^), complex anatomical scales (eg, Injury Severity Score^22^); systems requiring laboratory tests not available at the scene (eg, Emergency Trauma Score^23^); or scores requiring imaging studies (eg, Trauma-associated Severe Hemorrhage Score^24^). Finally, the selected scores included the RTS,^7^ NTS,^8^ MGAP,^9^ and BIG.^25^

## OUTCOMES

Primary outcome of the current study was LSI performed at the scene or en route. The following LSI were included in the analysis: invasive mechanical ventilation (conventional orotracheal intubation and videolaryngoscope in cases of difficult airway); and/or administration of tranexamic acid and/or noradrenaline (any dose). We did not include additional vasopressors as LSI (eg, dopamine, dobutamine or vasopressin) for the composite outcome because noradrenaline is the standard pharmacologic agent in ALS, in accordance with the internal procedure guidelines of our health system, which describe noradrenaline as the vasopressor of choice for hypovolemic shock unresponsive to volume with hemodynamic compromise. Neither did we include blood administration, since this procedure is conducted exclusively in the ED in our health system. As a secondary outcome we included unplanned ICU admission, and two-day in-hospital mortality (all cause).

Cases (ie, patients showing a confirmed positive outcome [LSI and/or ICU admission and/or two-day mortality]), were all re-checked by the study coordinator. We excluded from analysis cases with missing data (complete-case study).

### Predictors and Data Abstraction

Before starting the project, specific training was given to all the staff in the correct use of the data collection notebook, how to take blood samples, perform the analysis, and maintain and clean the devices used for variables collection, which are stated below. The study coordinator regularly visited all the ambulance stations.

Epidemiological variables (gender, age, urban or rural area, vector of transfer, and intervention times) were collected by an EMT, and baseline vital signs (respiratory rate, oxygen saturation, blood pressure, heart rate, temperature, and GCS), were collected by the ERN during the first contact of the ALS unit with the trauma patient. Pulse oxygen saturation, blood pressure, and pulse were determined with the LifePAK 15 monitor-defibrillator (Physio-Control, Inc, Redmond, WA), and temperature with the ThermoScan PRO 6000 thermometer (Welch Allyn, Inc, Skaneateles Falls, NY).

Subsequently, once the venous line was cannulated, a blood sample was extracted by the ERN for analysis of parameters, including creatinine, lactate, base excess, and INR. Blood analysis was conducted using the epoc Blood Analysis System (Siemens Healthcare GmbH, Erlangen, Germany) and CoaguChek Pro II System (Roche Diagnostics GmbH, Basel, Switzerland).

The ALS physician documented the application of LSI (invasive mechanical ventilation, and/or administration of tranexamic acid and/or noradrenaline), and the final prehospital diagnosis. Two days after the index event, a research associate from each hospital, collected the following hospital outcomes via electronic health records (EHR) review: hospital-inpatient, ICU admission, and two-day mortality (any cause).

### Statistical Analysis

All patient data was recorded electronically in a database created specifically for this purpose. Data was prospectively collected and registered in a database generated with the IBM SPSS Statistics for Apple version 20.0 (IBM Corp, Armonk, NY). The caseload entry system was test-run to delete unclear or ambiguous items and to verify the adequacy of the data-gathering system. To make a link between EMS medical records and the hospital’s EHR, an exact match was made with five of the following extractors: patient name; gender; age; day, arrival time, and incident code; and ambulance code and/or healthcare card number.

Data was never de-identified for the team responsible for data analysis. We assessed the descriptive results and the association between predictors and the outcome using the Mann-Whitney U test or the chi-squared test, as appropriate, and the effect size was provided as standardized mean difference. We used absolute values and percentages for categorical variables, and medians and interquartile ranges (IQR) for continuous variables. The a priori statistical plan was to use medians and IQRs if continuous variables were not normally distributed. Sample-size calculations were performed by assessing the power calculation based on a specific comparison using pwr package in R (The R Project for Statistical Computing, Vienna, Austria), considering a statistical power (from 1–100) of 97, a significance level of *P* = .001, and a mSOFA score difference between cases and non-cases of 84%. The sample used for training and validation, derived from a cross validation (ie, n/10), was n = 76.

Prior to score development and validation the sample was randomly split, preserving the proportion of the outcome variable, by using a 10-fold cross-validation, which has been used to overcome overfitting. (Further detail can be found in supplementary data p4.) The mSOFA score validation and calibration required a first step of fitting a logistic regression in which the score (as a continuous variable) was the predictive variable and the LSI, two-day mortality, or ICU admission the outcome. Considering the whole cohort, we plotted the observed distribution of the outcomes and a curve of the predicted probability of the outcome according to the mSOFA score, including the confidence interval (CI).

To assess the reliability of mSOFA and its comparison against other well-established scores, we evaluated all the scores in three different ways: with their discriminative power (assessed by the area under the receiver operating characteristic [ROC] curve and the area under the ROC curve [AUC]); their calibration (observed vs predicted outcome agreement; and with decision curve analysis [DCA], clinical utility). In particular, the mSOFA discrimination capacity was assessed by the AUC. The calibration was also performed by calculating the calibration curve, that is, plotting predicted vs observed probability of the outcome, and determining several metrics associated with calibration (explained below).

We assessed the discriminative power of mSOFA by ROC curve analysis and AUC, including 95% CIs, a *P*-value of the hypothesis testing (H0: AUC = 0.5). All 95% CIs were obtained by bootstrapping (2,000 iterations). We assessed further parameters of the ROC: specificity; sensitivity; positive predictive value; negative predictive value; positive likelihood ratio; and negative likelihood ratio. We also reported the maximum potential effectiveness achieved by the scores, with the Youden Index (in terms of sensitivity and specificity) serving also as a summary of the whole ROC curve. We compared AUCs using the Delong test.

We analyzed the goodness of fit of the model against the observed probability using different adjustments: logistic and nonparametric fit using LOWESS. We calculated several additional statistics: Somers’ D rank correlation; ROC area; Nagelkerke-Cox-Snell-Maddala-Magee R-squared index; discrimination index; unreliability index; the quality index; Brier score (average squared difference in p and y); intercept, slope, maximum absolute difference in predicted and loess-calibrated probabilities; the average of the previous parameter; the 0.9 of the previous parameter; and the Spiegelhalter Z-test for calibration accuracy and its two-tailed *P*-value.

We used DCA to compare mSOFA with those scores already used in clinical practice.

All statistical analyses were performed in R version 4.0.3 using packages described in supplementary data p4.

### Role of the Funding Source

The funder of the study had no role in study design, data collection, data analysis, data interpretation, or writing of the report. The corresponding authors had full access to all the data in the study and had final responsibility for the decision to submit for publication.

## RESULTS

### Patient Characteristics

In the current study, 763 patients from 44 ambulance stations (six ALS and 38 BLS) fulfilled the inclusion criteria (supplementary figure S1). Their median age was 52 years (IQR: 37–70 years), and 266 (34.9%) were female.

**Figure 1.**
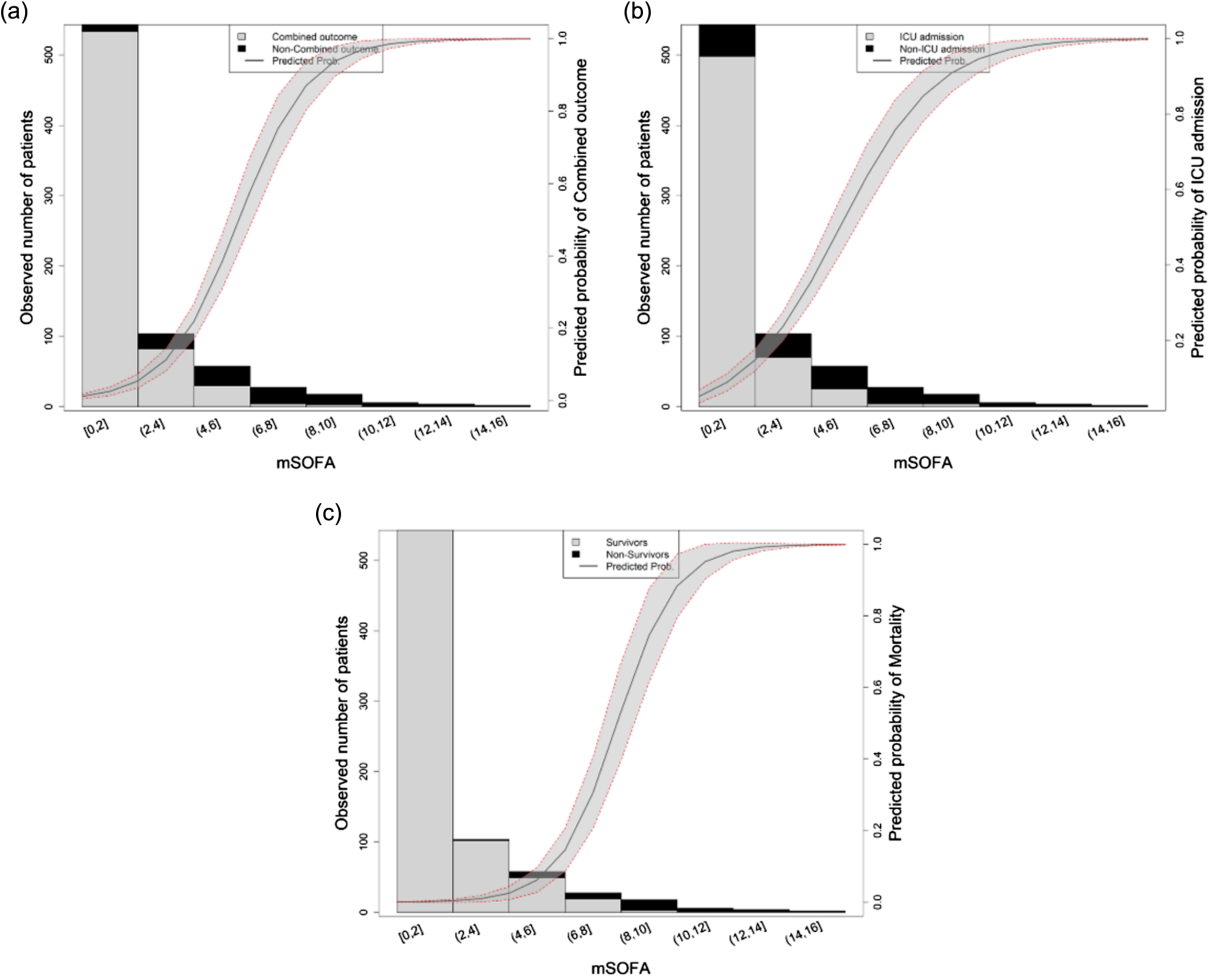
Probability of different outcomes based on the value of mSOFA in prehospital care: a) life-saving interventions; b) ICU admission; and c) two-day mortality. The solid line shows the predicted probability of the outcome; gray-shadowed area shows the 95% confidence interval. *mSOFA*, modified Sequential Organ Failure Assessment; *ICU*, intensive care unit.

The prehospital-LSI rate was 14.5% (111 cases), while 48 cases (6.3%) were treated with tranexamic acid, 28 (3.7%) with noradrenaline, and 92 cases (12.1%) with invasive mechanical ventilation. In 69 of the prehospital-LSI patients (62.2%), only one intervention was performed; two prehospital-LSI were performed in 27 patients (24.3%), and in 15 patients (13.5%) all three prehospital-LSI were required. Trauma patients subjected to prehospital LSI were predominantly male, 18–49 years, and polytraumatized (life-threatening involvement of two or more systems), with a remarkable rate of penetrating trauma (15.3%). Of the LSI group cases, 93.7% presented ICU admission (104 cases), and the two-day in-hospital mortality rate was 35.1% (39 cases). The mSOFA-associated variables showed statistically significant differences between cases with prehospital-LSI and non-LSI cases (*P* < .001) (see Table 1).

**Table 1.** Baseline clinical and biomarker characteristics of the study population.

	Total	Non-LSI	LSI	Standardized difference^b^	*P-*value^c^
No. (%) with data^a^	763 (100)	652 (85.5)	111 (14.5)	N.A.	N.A.
Age, years	52 (37–70)	52 (37–70)	50 (34–65)	0.086	.402
Age groups, years				0.101	.559
18–49	349 (45.7)	295 (45.2)	54 (48.6)		
50–74	269 (35.3)	232 (35.6)	37 (33.3)		
>75	145 (19)	125 (19.2)	20 (18)		
Gender, female	266 (34.9)	234 (35.9)	32 (28.8)	0.151	.149
ALS	507 (66.4)	401 (61.5)	106 (95.5)	0.909	<.001
Zone, urban	478 (62.6)	422 (64.7)	56 (50.5)	0.292	.004
Isochronous, min					
Arrival time	11 (8–16)	11 (8–16)	12 (10–23)	0.361	.001
Support time	31 (23–40)	30 (22–28)	34 (26–48)	0.423	<.001
Evacuation time	12 (8–20)	12 (8–19)	15 (10–28)	0.44	<.001
Basal vital signs					
RR, breaths/min	18 (14–21)	18 (14–20)	18 (12–27)	0.176	.147
SpO2 , %	97 (95–99)	97 (95–99)	93 (87–97)	0.894	<.001
FiO2 , %	0.21 (0.21–0.21)	0.21 (0.21–0.21)	0.21 (0.21–0.21)	0.608	.031
SaFi	462 (448–471)	462 (452–471)	429 (310–462)	0.952	<.001
SBP, mm Hg	133 (117–146)	134 (120–147)	121 (89–142)	0.523	<.001
DBP, mm Hg	80 (68–90)	80 (70–90)	66 (55–87)	0.508	<.001
MBP, mm Hg	97 (85–108)	98 (88–108)	86 (77–104)	0.543	<.001
HR, beats/min	84 (71–100)	83 (70–98)	92 (77–118)	0.521	<.001
Temperature, °C	36 (35.7–36.4)	36 (35.8–36.4)	35.8 (34.8–36.1)	0.474	<.001
GCS, points	15 (15–15)	15 815–15)	9 (5–14)	1.634	<.001
Lactate, mmol/L	2.42 (1.64–3.54)	2.16 (1.45–3.09)	5.67 (3.25–8.71)	1.158	<.001
Creatinine, mgr/dL	0.86 (0.74–1.09)	0.84 (0.73–1.01)	1.09 (0.87–1.68)	0.616	<.001
BE, mEq/L	0.6 (−2.6;−1.8)	0.8 (−1.6;1.9)	−4.6 (−10;−1.5)	1.143	<.001
INR	1 (1–1.1)	1 (1–1.1)	1 (1–1.1)	0.228	.057
mSOFA, points	1 (0–3)	1 (0–2)	6 (4–8)	1.994	<.001
Trauma mechanism				0.419	<.001
Penetrating	39 (5.1)	22 (3.4)	17 (15.3)		
Blunt	724 (94.9)	630 (96.6)	94 (84.7)		
Trauma type				0.343	<.001
Polytraumatized	106 (13.9)	53 (8.1)	53 (47.7)		
Polycontused	83 (10.9)	83 (12.7)	0		
Head and neck	257 (33.7)	221 (33.9)	36 (32.4)		
Thorax	52 (6.8)	47 (7.2)	5 (4.5)		
Abdomen-pelvic	32 (4.2)	25 (3.8)	7 (6.3)		
Spinal	46 (6)	45 (6.9)	1 (0.9)		
Orthopedic	156 (20.4)	153 (23.5)	3 (2.7)		
Burns	31 (4.1)	25 (3.8)	6 (5.4)		
Hospital outcomes					
Hospital-inpatient	372 (48.8)	265 (40.6)	107 (94.9)	1.501	<.001
ICU admission	162 (21.2)	58 (8.9)	104 (93.7)	3.204	<.001
2-day mortality	47 (6.2)	8 (1.2)	39 (35.1)	0.979	<.001

*LSI*, life-saving interventions; *NA*, Not applicable; *ALS*, Advanced Life Support; *RR*, respiratory rate; *SpO*2 , oxygen saturation; *FiO*2 , fraction of inspired oxygen; *SaFi*, pulse oximetry saturation/fraction of inspired oxygen ratio; *SBP*, systolic blood pressure; *DBP*, diastolic blood pressure; *MBP*, mean blood pressure; *HR*, heart rate; *GCS*, Glasgow coma scale; *BE*, base excess; *INR*, International normalized ratio; *mSOFA,* modified Sequential Organ Failure Assessment; *ICU*, intensive care unit.

a Values expressed as total number (fraction) and medians [25^th^ percentile-75^th^ percentile], as appropriate.

b The Cohen d-test was used for estimated effect size.

c The Mann-Whitney U test, *t*-test, or chi-squared test was used as appropriate.

### mSOFA Validation

The validation of the mSOFA score was performed by ROC analysis assessing AUC values. The AUCs were as follows: 0.927 (95% CI 0.898–0.957) for LSI; 0.845 (95% CI 0.808–0.882) for ICU admission; and 0.979 (95% CI 0.966–0.991) for two-day mortality. Further details on the model performance can be found in supplementary tableS2. As can be observed in Figure 1, the predictive probability for each outcome as a function of mSOFA presented a typical sigmoid curve, meaning that the number of cases (for all the outcomes) increased with increasing mSOFA values. Curves were steeper for LSI and ICU, indicating that the mSOFA value at which the cases increased for this outcome was lower than that for mortality.

Finally, the calibration results showed that the best performance of mSOFA was for mortality according to Brier, resulting in 0.06 (95% CI 0.063–0.067) for LSI; 0.11 (95% CI 0.110–0.115) for ICU; and 0.03 (95% CI 0.019–0.049) for mortality. However, when considering the slope or the calibration-in-the-large (or intercept), the best performance was for ICU, followed by LSI and mortality (in that order). Results of slope were 0.98 (95% CI 0.73–1.23) for LSI; 1.00 (95% CI 0.86–1.15) for ICU; and 0.89 (95% CI 0.48–1.30) for mortality, while those of calibration-in-the-large were 0.02 (95% CI −0.39–0.45) for LSI; 0.004 (95% CI −0.24–0.25) for ICU; and −0.50 (95% CI −1.82–0.82) for mortality.

### mSOFA vs Other Scoring Systems

**Figure 2.**
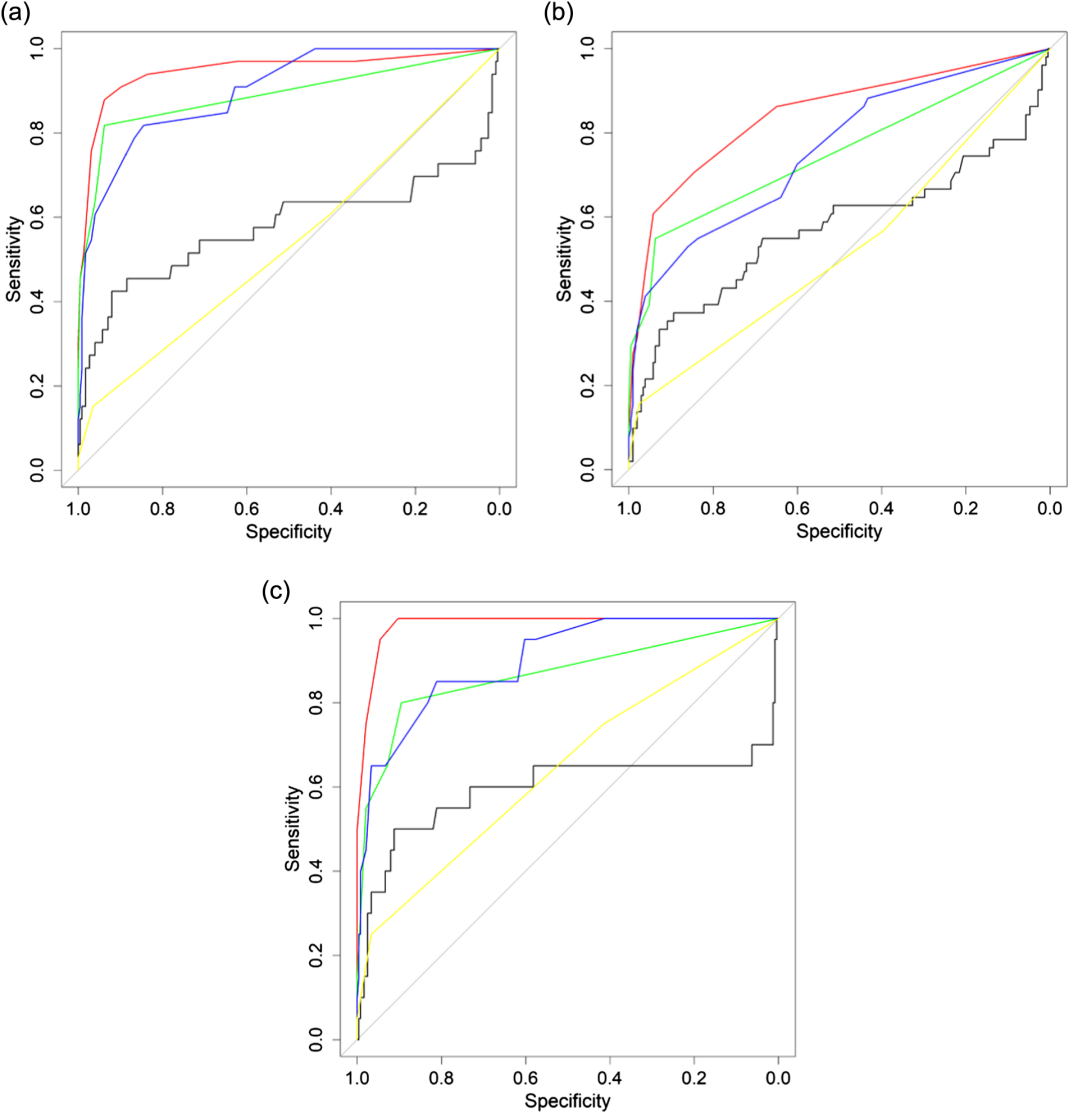
Discrimination analysis results of each model. Discrimination capacity of the scores was assessed by the area under the receiver operating characteristic (ROC) curve (AUC) for a) life-saving interventions; b) intensive care unit (ICU) admission; and c) two-day mortality. Red line = modified Sequential Organ Failure Assessment (mSOFA); green line = revised trauma score (RTS); blue line = combination of mechanism, Glasgow Coma Scale, age, and arterial pressure score (MGAP); black line = BIG score; yellow line = new trauma score (NTS).

**Figure 3.**
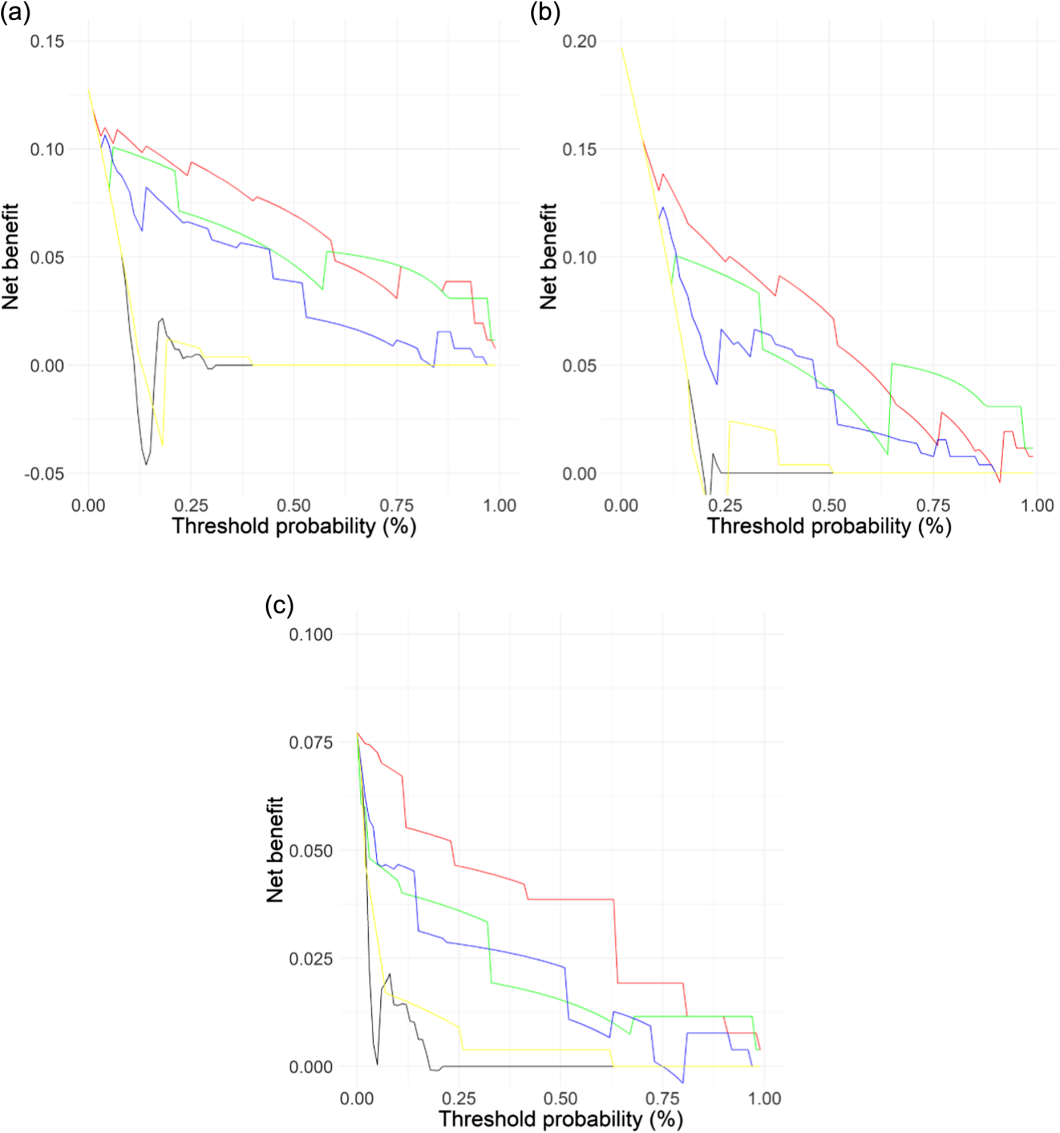
Decision curve analysis results of each model: a) life-saving interventions; b) intensive care unit admission; and c) two-day mortality. Red line = modified Sequential Organ Failure Assessment; green line = revised trauma score); blue line = combination of mechanism, Glasgow Coma Scale, age, and arterial pressure score; black line = BIG score; yellow line = New Trauma Score. *BIG score*, admission base deficit + international normalized ratio + Glasgow Coma Scale.

The comparison between mSOFA and the other scores was assessed by three different procedures. First, the discrimination power using the AUC comparison showed that mSOFA outperformed the other scores for all the outcomes evaluated (Figure 2); this was corroborated by the results from the Delong test (Supplementary tableS3) in which mSOFA presented statistically significant differences vs all the scores for all outcomes (*P*-value ranged between *P* < .01 and *P* < .001, except for LSI in which *P* = .078 vs RTS, and *P* = .061 vs MGAP). Secondly, similarly to discrimination analysis, DCA (Figure 3) showed a higher net benefit for mSOFA than other scores throughout all the threshold probability and for all outcomes, with the exception of the higher threshold, in which the net benefit was similar for LSI or lower for ICU admission compared to RTS.

Thirdly, the calibration-derived metrics showed that mSOFA presented the best performance for LSI. For instance, the Brier score of mSOFA was the lowest compared to the other scores for all the evaluated outcomes. Interestingly, when considering the fitted calibration curves, either logistic or nonparametric (LOWESS), mSOFA presented the best fit. Further details on the calibration results can be found in supplementary FigureS4.

## DISCUSSION

To our knowledge, this is the first study to analyze the predictive ability of prehospital mSOFA scores to detect high-risk trauma patients at the scene or en route. The mSOFA score presented an excellent AUC for prediction of prehospital LSI, unplanned ICU admissions, and two-day in-hospital mortality, outperforming all the scores analyzed.

The X-A-B-C-D-E resuscitation guidelines clearly outline the priorities in primary assessment and initial care of major trauma.^26^ Upon identification and control of external bleeding hemorrhage, the next life-threatening priority is to ensure airway patency and/or ineffective ventilation, coupled with quick recognition of subtle signs of shock at early onset that allow rapid treatment start. Considering the above, in the first moments post-injury, EMS personnel should prioritize the rapid identification and response to hypovolemic shock of hemorrhagic origin as a top priority, together with initial airway management on scene.^27^^,^^28^

Several scores have been developed to forecast the severity of a trauma patient’s condition; however, studies evaluating the predictive ability of different scores to determine prehospital LSI requirement are scarce. Galvagno et al^11^ analyzed the performance of venous lactate to predict prehospital LSI, showing an AUC of 0.71. Radowsky et al^29^ examined the ability of the prehospital, handheld, tissue oximeter to identify occult shock, with poor results (AUC = 0.51). Liu et al^30^ studied the capability of different standard vital signs (heart rate, lower systolic blood pressure, shock index, pulse pressure, and GCS components), while Kumar et al^31^ analyzed the feasibility to predict LSI of a model based on heart rate variability. In both cases overall results were modest (AUC = 0.72 and 0.75, respectively). In summary, isolated studies have analyzed standard vital signs and/or trauma scores, reporting AUCs lower than that of the mSOFA score.

The mSOFA is a five-parameter system assessable in prehospital care from the first contact with patients,^18^ which allows the identification of potential high-risk trauma patients. This scoring system includes respiratory (SaFi), hemodynamic (MAP), neurological (GCS), and oxidative metabolism (creatinine and lactate) endpoints, providing a global overview of the trauma patient’s status.

## LIMITATIONS

While the strengths of the present study are the large number of EMS records we examined over a long period of time and the use of standardized procedures for the management of trauma patients, as well as the homogeneous training of all EMS personnel involved, the study does have several limitations. First, the data was not blinded. To minimize bias, data was collected continuously 24 hours a day throughout the year, and ambulances served both urban and rural areas, involving different EDs. In addition, EMS personnel did not know the scores analyzed or their interpretation or calculated risks, and the outcomes were not known to hospital investigator associates. As a double-check setup, the study coordinator audited all notified patients with a primary positive outcome. Second, some of the scores tested required the use of prehospital POCT, which requires a certain training and is not widely implemented in EMS. Third, even though thoracic decompression techniques are considered to be LSI, they were not included in the study nor was there exact data on the crystalloid volume administered. (In future studies these inputs will be recorded to improve predictive capacity and to improve the identification of high-risk patients.)

Fourth, the ongoing study started before the current COVID-19 pandemic and was in progress, continuously adding cases into the database. The potential long-term consequences of COVID-19 are yet to be determined; thus, it remains unclear what the effect of this new pathology has on patients who have already suffered the disease or its long-term sequelae. Larger studies are required to understand the impact of the pandemic on medical emergency care, and specifically on trauma patients. Fifth, the interpretation of the DCA and calibration results should be interpreted from a qualitative point of view, rather than from a quantitative/statistical point of view. They are intended to support the AUC findings rather than be interpreted alone. Finally, the current study was developed in a single country, with particular conditions of the Spanish health system, such as POCT availability in the EMS. The generalizability of this study will require further studies in different health systems.

## CONCLUSION

The mSOFA score can help EMS personnel recognize high-risk patients. The identification of three key outcomes—life-saving interventions on scene, unplanned ICU admission, and two-day mortality—can play a critical role in better management of these rapidly evolving and complex cases.

## Supplementary Information








