# Supplementary material for: Prehospital mSOFA Score for Quick Prediction of Life-Saving Interventions and Mortality in Trauma Patients: A Prospective, Multicenter, Ambulance-based, Cohort Study
Source: West J Emerg Med. 2023 Aug 8;24(5):868–77. doi: 10.5811/westjem.59048 (PMC10527847; doi:10.5811/westjem.59048)
Supplement: Supplementary file 1 [file wjem-24-868-s001.docx]

**Supplementary Data**

Supplementary Data

Table of contents

Supplementary methods…………………………………………………………………3

Transparent Reporting of a multivariable prediction model for Individual Prognosis Or Diagnosis (TRIPOD) guidance...……………………………….…3

Sample size……………………………………………………………………....4

Software….……………………………………………………………………....4

Cross validation………………………………………………………………….4

Validation ………………………………………………………………………..4

Supplementary Results…………………………………………………………………..5

- Supplementary figure S1: Flowchart.…………………………………………..5

- Supplementary Table S2: Further parameters of ROC curve analysis of mSOFA for different outcomes……………………………………………..…...6

- Supplementary Table S3: AUC comparison between different scores for the three different outcomes ………………………………….………………....…..7

- Supplementary figure S4a: Calibration of each score for life-saving interventions for a) mSOFA, b) RTS, c) MGAP, d) BIG score, e) NTS.………..8

- Supplementary figure S4b: Calibration of each score for ICU admission for a) mSOFA, b) RTS, c) MGAP, d) BIG score, e) NTS.………………………...…..9

- Supplementary figure S4c: Calibration of each score for 2-day mortality for a) mSOFA, b) RTS, c) MGAP, d) BIG score, e) NTS.……………………...……10

References.......................................................................................................................11

**Supplementary Methods**

This study is in accordance with the Transparent Reporting of a multivariable prediction model for Individual Prognosis Or Diagnosis (TRIPOD) guidance^1^. The following image shows the page number with each TRIPOD item.


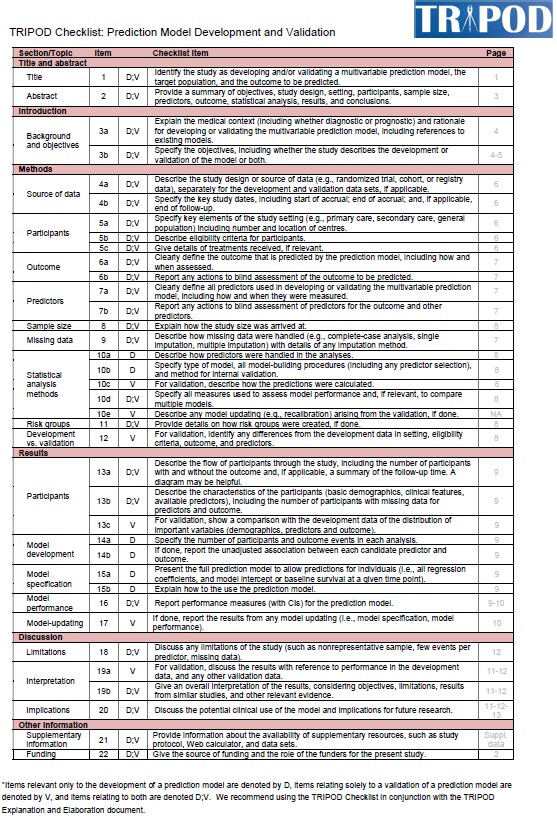


***Sample Size***

Based on previous studies^2^, the statistical power (from 1 to 100) of the present study for each individual score is equal to 97.1 based on the following considerations: (i) the sample used for training and validation is derived from a cross validation, i.e. n/10, which it is equal to n = 76, (ii) significant level of p = 0.001, (iii) an mSOFA difference between cases and non-cases of 84%.

***Software***

All calculations and analyses were performed by using our own codes, R packages and base functions in R, version 4.0.3 (http://www.R-project.org; the R Foundation for Statistical Computing, Vienna, Austria). In particular, the following packages were used: caret (version 6.0-86)^3^ for cross validation, pROC (version 1.16.2)^4^, for C-statistic calculations, rms (version 6.2-0)^5^ for calibration metrics calculation.

***Cross validation***

The ten-fold cross-validation process consists in a split of the data in training and validation cohorts, this is performed randomly and preserving the outcome distribution in both cohorts. This process was performed ten times to avoid the overfitting of the model^6^

***Validation***

In particular, the mSOFA discrimination capacity was assessed by the area under the receiver operating characteristic (ROC) curve (AUC). The calibration was also performed by calculating the calibration curve, that is, plotting predicted vs observed probability of the outcome, and determining several metrics associated to calibration (explained below).

The discriminative power of mSOFA was assessed by ROC curve analysis and AUC, including the 95% confidence intervals (CI) and the p value of the hypothesis testing (H0: AUC=0.5). All 95% CI were obtained by bootstrapping (2000 iterations). Further parameters of the ROC were assessed: specificity (sp), sensitivity (sen), positive predictive value, negative predictive value, positive likelihood ratio, and negative likelihood ratio. The maximum potential effectiveness achieved by the scores, the Youden Index (in terms of sensitivity and specificity, serving also as a summary of the whole ROC curve) were also reported. AUCs were compared by the Delong’s test.

The calibration^7^ of the score, which represents the predicted vs observed probability of the outcome, should be as close to the diagonal as possible to rule out possible over- or under-estimation of the evaluated score (predicted probability) as compared to the ground truth (observed probability). The goodness of fit of the model against the observed probability (grey diagonal) was analyzed by using different types of adjustments: logistic (solid line) and nonparametric (dashed line). Additionally several statistics are calculated: Somers' D rank correlation (Dxy), ROC area (C), Nagelkerke-Cox-Snell-Maddala-Magee R-squared index (R2), Discrimination index D (D), Unreliability index (U), the quality index (Q), Brier score (average squared difference in p and y) (Brier), Intercept, Slope, maximum absolute difference in predicted and loess-calibrated probabilities (Emax), the average of the previous parameter (Eavg), the 0.9 of the previous parameter (E90), the Spiegelhalter Z-test for calibration accuracy (S:z), and its two-tailed P-value (S:p).

The decision curve analysis (DCA) allows to compare prediction models, it was performed by using the code from Vickers AJ et al.^8,9^. In particular, DCA deals with the clinical consequences of using new models as compared to those already used in clinical practice. Through the calculations of net benefit (of using a particular model compared to another) weighted by the odds of the outcome at a given threshold probability allows to plot benefit (y-axis) against preference (x-axis).

**Supplementary Results**

**Flowchart**

Study #01 (ISRCTN48326533)

January-December 2020

Study #02 (ISRCTN49321933)

January 2021-April 2022

Screened (n=1212)

Excluded (n=365)

♦ Not fulfilled inclusion criteria (n=331)

♦ Declined to participate (n=6)

♦ Failed to complete consent form) (n=28)

Participated (n=847)

Refusals (n=84)

♦ Excluded by duplication (n=11)

♦ Missing data (n=29)

♦ Lost to follow-up (n=44)

Analyzed (n=763)

**Supplementary Table S2: Further parameters of ROC curve analysis of mSOFA for different outcomes.** a) life-saving interventions, b) ICU admission, and c) 2-day mortality.

Note: the columns including “youden”, refer to the maximum potential effectiveness achieved by the scores, i.e., the Youden Index, in terms of sensitivity and specificity, and the threshold at which these values are achieved.

| a) |  | |  |  | |  |  |  |  |  |  |
| --- | --- | --- | --- | --- | --- | --- | --- | --- | --- | --- | --- |
|  | Sp | sen | | ppv | npv | | plr | nlr | threshold (youden) | sp (youden) | sen (youden) |
| mSOFA | 90.99 (88.26-  93.71) | 41.37 (35.70-  47.04) | | 78.67 (74.52-82.82) | 91.06 (90.27-91.85) | | 56.12 (49.44-62.79) | 0.59 (0.54-0.65) | 2.7 (2.39-3.00) | 83.16 (80.93-85.45) | 89.59 (87.59-91.58) |

*Abbreviations*: see below

| b) |  | |  |  | |  |  |  |  |  |  |
| --- | --- | --- | --- | --- | --- | --- | --- | --- | --- | --- | --- |
|  | Sp | sen | | ppv | npv | | plr | nlr | threshold (youden) | sp (youden) | sen (youden) |
| mSOFA | 91.04 (88.82-  93.99) | 31.91 (27.02-  36.79) | | 80.55 (76.95-84.14) | 84.44 (83.55-85.33) | | 50.53 (43.61-57.45) | 0.70 (0.65-0.75) | 2.5 (2.5-2.5) | 82.86 (82.50-83.22) | 72.22 (71.55-72.88) |

*Abbreviations*: see below

| c) |  | |  |  | |  |  |  |  |  |  |
| --- | --- | --- | --- | --- | --- | --- | --- | --- | --- | --- | --- |
|  | Sp | sen | | ppv | npv | | plr | nlr | threshold (youden) | sp (youden) | sen (youden) |
| mSOFA | 89.17 (85.68-92.66) | 56.53 (49.26-63.81) | | 67.01 (60.51-73.50) | 97.28 (96.83-  97.72) | | 61.09 (52.13-  70.05) | 0.51 (0.45-0.57) | 4.78 (4.33-5.23) | 97.04 (89.27-92.80) | 95.25 (93.98-96.52) |

*Abbreviations*: sp: specificity; sen, sensitivity; ppv: positive predictive value; npv: negative predictive value; plr: positive likelihood ratio; npr: negative likelihood ratio. Values between parenthesis refer to 95% confidence interval.

**Supplementary Table S3: AUC comparison between different scores for the three different outcomes.** a) life-saving interventions, b) ICU admission, and c) 2-day mortality.

The table shows the p-values (Delong’s test) of each comparison. The diagonal (bold values) shows the AUC and 95% confidence interval.

| a) |  | |  |  | |  |  |
| --- | --- | --- | --- | --- | --- | --- | --- |
|  | mSOFA | RTS | | MGAP | BIG score | | NTS |
| mSOFA | **0.927 (0.898-0.957)** |  | |  |  | |  |
| RTS | 0.078 | **0.889 (0.819-0.959)** | |  |  | |  |
| MGAP | 0.061 | 0.667 | | **0.900 (0.844-0.955)** |  | |  |
| BIG score | <0.001 | <0.001 | | <0.001 | **0.579 (0.4357-0.722)** | |  |
| NTS | <0.001 | <0.001 | | <0.001 | 0.599 | | **0.539 (0.434-0.644)** |

| b) |  | |  |  | |  |  |
| --- | --- | --- | --- | --- | --- | --- | --- |
|  | mSOFA | RTS | | MGAP | BIG score | | NTS |
| mSOFA | **0.845 (0.808-0.882)** |  | |  |  | |  |
| RTS | 0.002 | **0.748 (0.677-0.820)** | |  |  | |  |
| MGAP | 0.025 | 0.757 | | **0.757 (0.679-0.835)** |  | |  |
| BIG score | <0.001 | 0.014 | | 0.006 | **0.572 (0.464-0.680)** | |  |
| NTS | <0.001 | <0.001 | | 0.001 | 0.439 | | **0.522 (0.438-0.610)** |

| c) |  | |  |  | |  |  |
| --- | --- | --- | --- | --- | --- | --- | --- |
|  | mSOFA | RTS | | MGAP | BIG score | | NTS |
| mSOFA | **0.979 (0.966-0.991)** |  | |  |  | |  |
| RTS | 0.013 | **0.867 (0.771-0.963)** | |  |  | |  |
| MGAP | 0.017 | 0.332 | | **0.901 (0.832-0.970)** |  | |  |
| BIG score | <0.001 | 0.028 | | 0.009 | **0.593 (0.397-0.789)** | |  |
| NTS | <0.001 | 0.008 | | 0.001 | 0.625 | | **0.643 (0.515-0.772)** |

**Supplementary figure S4a: Calibration of each score for life-saving interventions.** a) mSOFA, b) RTS, c) MGAP, d) BIG score, e) NTS.

**
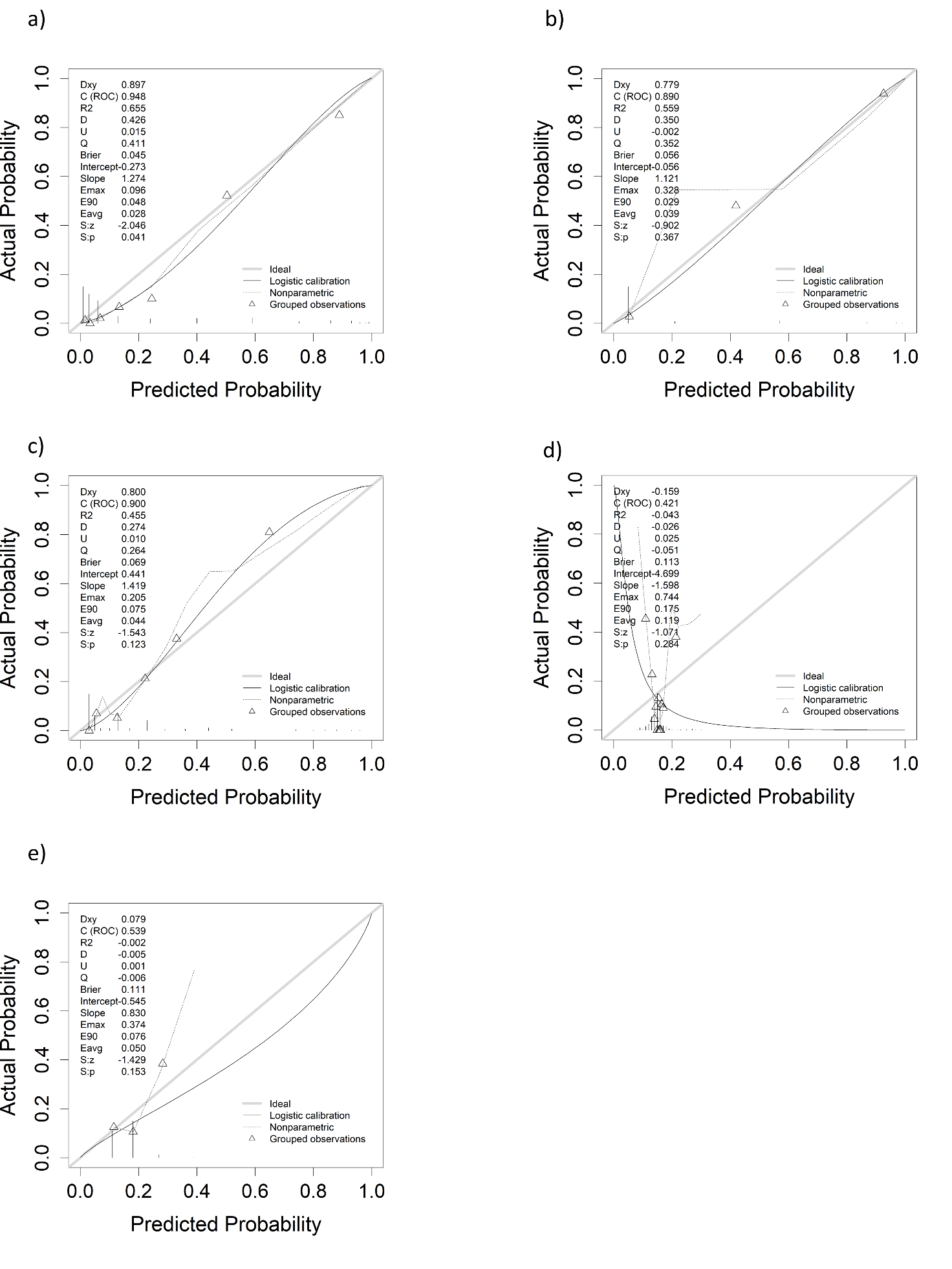
**

**Supplementary figure S4b: Calibration of each score for ICU admission.** a) mSOFA, b) RTS, c) MGAP, d) BIG score, e) NTS.

**
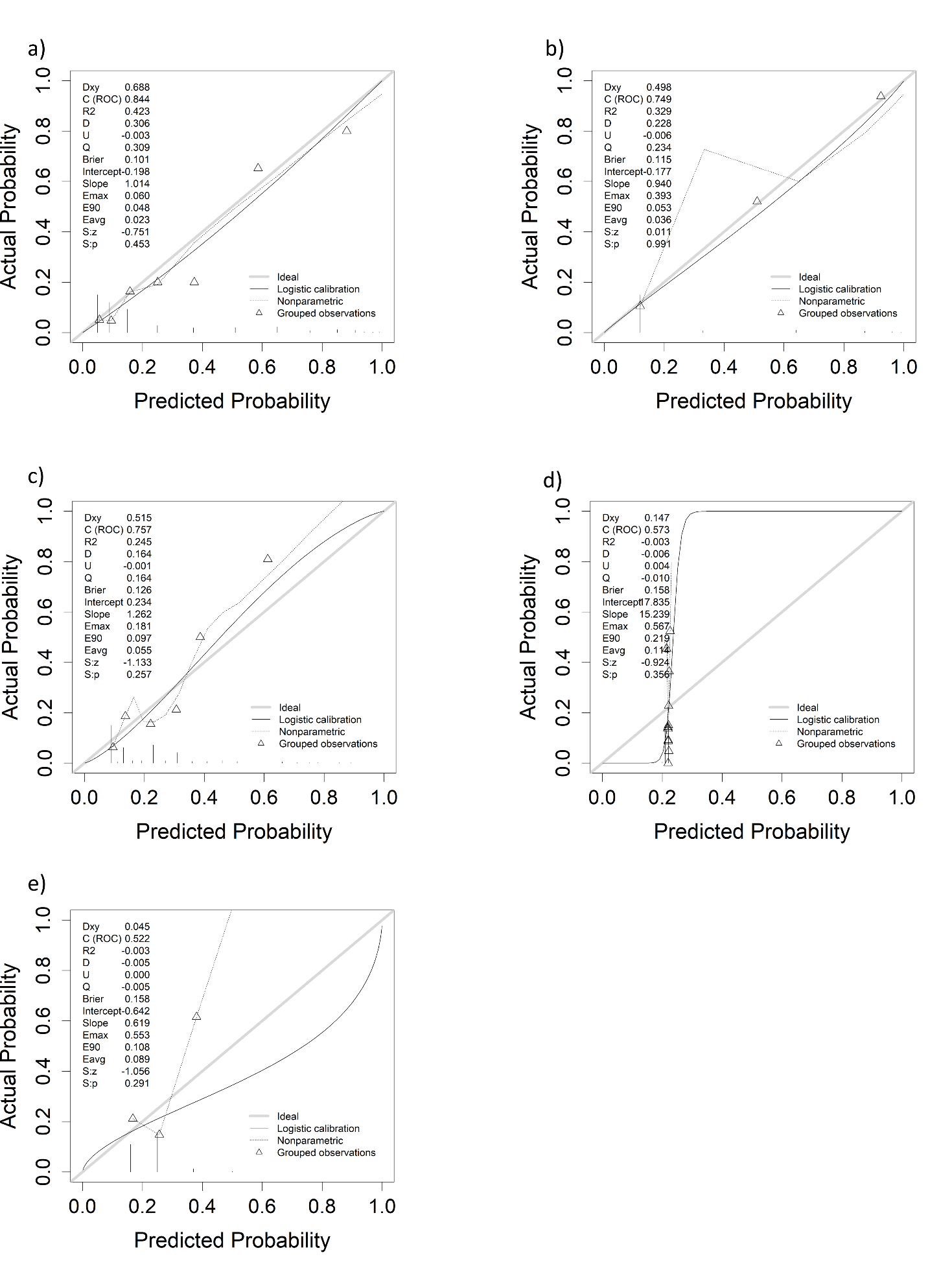
**

**Supplementary figure S4c: Calibration of each score for 2-day mortality.** a) mSOFA, b) RTS, c) MGAP, d) BIG score, e) NTS.

**
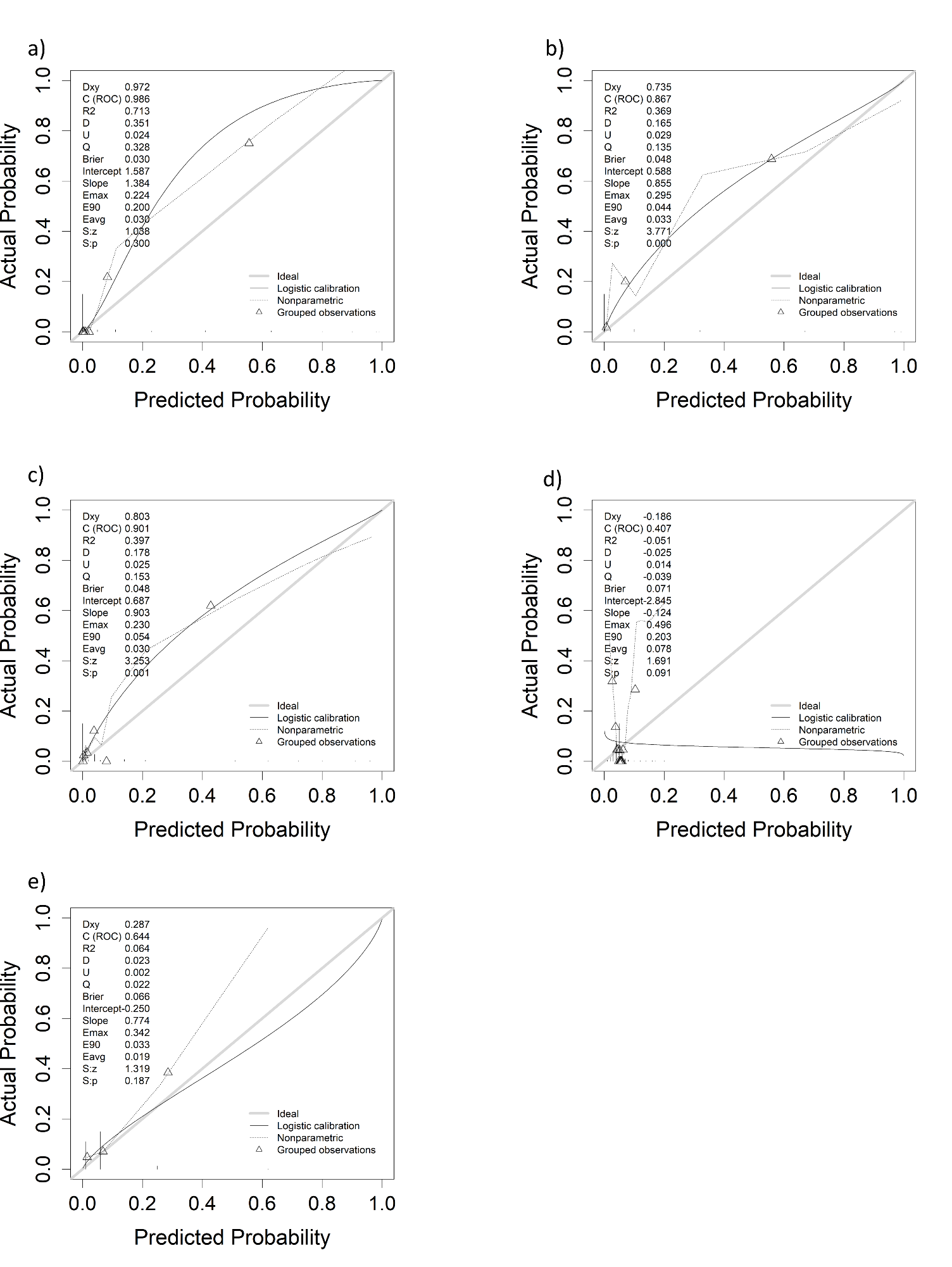
**

**References**

1. Collins GS, Reitsma JB, Altman DG, Moons KGM. Transparent reporting of a multivariable prediction model for individual prognosis or diagnosis (TRIPOD): the TRIPOD statement. BMJ 2015; 350: g7594.

2. Martín-Rodríguez, F., Sanz-García, A., Del Pozo Vegas, C., Ortega, G. J., Castro Villamor, M. A., & López-Izquierdo, R. (2021). Time for a prehospital-modified sequential organ failure assessment score: An ambulance-Based cohort study. The American journal of emergency medicine, 49, 331–337.

3. Kuhn, M. Building Predictive Models in R Using the caret Package. Journal of Statistical Software 2008, 28(5), 1–26.

4. Robin X, Turck N, Hainard A, et al. pROC: an open-source package for R and S+ to analyze and compare ROC curves. BMC Bioinformatics 2011; 12: 77.

5. Harrell Jr FE. rms: Regression Modeling Strategies. 2019.

6. Han H, Jiang X: Overcome Support Vector Machine Overfitting in Diagnosis. Cancer Informatics 2014; 13:145–58

7. Alba AC, Agoritsas T, Walsh M, et al. Discrimination and Calibration of Clinical Prediction Models: Users' Guides to the Medical Literature. JAMA. 2017 Oct 10;318(14):1377-1384.

8. Vickers AJ, Elkin EB. Decision curve analysis: a novel method for evaluating prediction models. Medical Decision Making. 2006 Nov-Dec;26(6):565-74.

9. https://www.mskcc.org/departments/epidemiology-biostatistics/biostatistics/decision-curve-analysis
